# Factors associated with parental anxiety in elective pediatric surgery

**DOI:** 10.3389/fmed.2026.1903817

**Published:** 2026-09-25

**Authors:** Ana Vlajkovic Ivanovic, Bojana Matejic, Andja Cirkovic, Miodrag Milenovic, Marija Stevic, Branislav Trifunovic, Jelena Pjevalica Dragic, Milica Pejovic Milovancevic

**Affiliations:** 1 Department of Anesthesiology and Intensive Therapy, University Children’s Hospital, Belgrade, Serbia; 2 Institute of Social Medicine, Faculty of Medicine, University of Belgrade, Belgrade, Serbia; 3 Institute for Medical Statistics and Informatics, Faculty of Medicine, University of Belgrade, Belgrade, Serbia; 4 Faculty of Medicine, University of Belgrade, Belgrade, Serbia; 5 Department of Anesthesiology, Emergency Center, University Clinical Center of Serbia, Belgrade, Serbia; 6 Department of Plastic and Reconstructive Surgery, University Children’s Hospital, Belgrade, Serbia; 7 Department of Cardiac Surgery, University Children’s Hospital, Belgrade, Serbia; 8 Department of Psychiatry, Institute for Mental Health, Belgrade, Serbia

**Keywords:** elective surgery, health literacy, parental anxiety, parents, pediatric surgery, preoperative anxiety

## Abstract

**Background:**

Parental perioperative anxiety is another critical aspect during the challenging times before the child's operation. The primary aim of this study was to examine factors associated with parental anxiety in the preoperative setting. Secondary aims were to compare anxiety levels between parents of surgical and non-surgical pediatric patients and to assess the internal consistency and construct validity of the State–Trait Anxiety Inventory (STAI) scales in this population.

**Methods:**

In this comparative cross-sectional study we included 196 parents. A total of 98 parents from the surgical group (84% mothers and 16% fathers) and 98 participants from the non-surgical group (74% mothers and 26% fathers) completed questionnaires aimed at assessing anxiety (State-Trait Anxiety Inventory-STAI, Form Y), functional health literacy (Test of Functional Health Literacy in Adults Short Form-STOFHLA) and general questionnaire specifically designed for this research.

**Results:**

Greater healthcare satisfaction was associated with lower, and higher ASA score and greater overall concern were associated with higher STAI-S scores in the surgical group, while parent`s age 30–40 years and good or very good household material condition were associated with lower STAI-S scores in the non-surgical group. The proportion of parents with higher levels of anxiety (STAI-S > 50) in the surgical and non-surgical group was 54% vs. 10%, respectively. Multivariable modeling confirmed that primary and secondary educational level, insufficient information regarding child`s health, obtaining information regarding child's health through conversations with friends/acquaintances who are not health workers, and higher concern score were associated with the presence of higher anxiety among parents whose children were scheduled for surgical intervention. In the non-surgical group only the presence of a serious medical condition in another child was related to lower anxiety (OR = 0.063, 95%CI OR = 0.01–0.38, *p* = 0.002). Internal consistency was excellent for both STAI-State and STAI-Trait scales (Cronbach's *α* > 0.90), while exploratory factor analysis supported the construct validity of the instrument confirming the expected two-factor structure.

**Conclusions:**

Primary and secondary educational level, insufficient information regarding child`s health, obtaining information from informal sources, and higher concern score were associated with the presence of higher anxiety among parents whose children were scheduled for surgical intervention.

## 1 Introduction

Preoperative anxiety is commonly defined as discomfort or tension arising from the anticipation of surgery, anesthesia, and hospitalization, and may be related to fear of pain, loss of control, and separation from loved ones (1, 2). Reported prevalence of preoperative anxiety varies widely across studies, reaching up to 92%, depending on the population, definition, and measurement instrument used (3).

The psychological well-being of children undergoing diagnostic and surgical interventions gained increased attention in the 1980s, leading to greater emphasis on parental presence in hospitals and their involvement in perioperative care (4). The perioperative period is particularly demanding for both children and parents and often triggers considerable psychological distress. Parental perioperative anxiety is another critical aspect during the challenging times before the child's operation. Parents commonly fear the uncertainty of the surgical outcome, worry about potentially life-threatening complications from surgery or anesthesia, and are concerned about the possibility of their child enduring pain, fear, or lasting disability (5–7).

Research has consistently demonstrated that the anxiety experienced by parents not only affects their own well-being, but directly correlates with the anxiety levels of their children undergoing surgery (8, 9) and even can be singled out as factor independently associated with the development of preoperative anxiety in a child (10–12). Furthermore, parental preoperative anxiety can be a factor that contributes to the development of child behavioral disorders that can persist for six months and even up to a year postoperatively (5, 13). Also, the relationship between the emotional state of a parent and a child is reciprocal; an anxious parent can contribute to the development of anxiety in a child, and the child's anxiety can also influence the parent's emotional state (10), which creates a vicious circle.

In addition, research suggests that parental preoperative anxiety may extend beyond the individual, affecting family dynamics, interaction with healthcare personnel, and the overall perioperative process, including cooperation and understanding of medical information (14). These facts highlight the importance of addressing parental anxiety as part of the perioperative care process. Furthermore, interventions aimed at reducing parental anxiety may contribute to more favourable outcomes in children, including decreased postoperative behavioral issues and improved recovery experiences (14–16).

The prevalence of parental anxiety related to their children's elective surgical procedures ranges from 44% (study by French authors) to 80% (study by Greek authors) (17, 18). Nevertheless, comparing results requires caution due to differences in assessment methods, research instruments, and the choice of study population (19–21).

Previous studies have several domains of factors associated with parental preoperative anxiety. Parent-related factors include sociodemographic characteristics such as parental age and gender, while child- and procedure-related factors include younger child age, severity of the child's condition, duration of hospitalization, and characteristics of the surgical procedure. Higher anxiety levels have been reported among mothers than fathers in some studies, although findings regarding parental gender are not consistent (6, 11, 22, 23). Communication- and information-related factors, including health literacy and the provision and sources of perioperative information, may also be relevant and are discussed below.

Functional health literacy may be particularly relevant in the perioperative setting because it influences parents' ability to understand medical instructions, appraise health information, and communicate with healthcare professionals. Lower parental health literacy has been associated with higher preoperative anxiety and difficulties in understanding and implementing medical recommendations (18, 24, 25). At the same time, the effect of perioperative information on anxiety may depend not only on the amount of information provided, but also on its source, content, timing, and comprehensibility. Professional sources may provide structured and clinically relevant information, whereas informal sources, including acquaintances and online content, may vary in credibility and quality. Previous studies also indicate that parents frequently report unmet information needs and that different types of perioperative information may have different effects on anxiety (6, 14, 17–19, 26).

Although parental preoperative anxiety has been widely studied, limited evidence is available from studies that consider sociodemographic characteristics, functional health literacy, and sources of health-related information within the same analysis. In addition, most previous studies have focused on parents of children undergoing surgery, without a non-surgical comparison group that could provide a reference for distinguishing anxiety associated with the preoperative context from anxiety occurring in routine pediatric care. Considering these domains within the same study and including a non-surgical pediatric comparison group may therefore provide a more comprehensive understanding of factors associated with parental anxiety in the preoperative setting.

In light of these considerations, we conducted a comparative cross-sectional study of parents of children scheduled for elective surgery and parents of children attending routine pediatric consultations. The primary aim was to examine factors associated with parental anxiety in the preoperative setting, including sociodemographic characteristics, functional health literacy, and sources of health-related information. A secondary aim was to compare anxiety levels between the surgical and non-surgical groups. As an additional methodological analysis, we examined the internal consistency and construct structure of the STAI scales in this study sample to assess their performance in the specific study context.

## 2 Materials and methods

### 2.1 Study design and settings

This study employed a comparative cross-sectional design (62). The research was conducted during a three-month period (March 2023-June 2023) at the University Children's Clinic and one public primary health care institution in the capital city of Serbia, Belgrade. The study was approved by the Ethics Committee of the Faculty of Medicine, University of Belgrade (No. 1322/XII-3, 30 December 2021), and was conducted in accordance with the principles of the Declaration of Helsinki.

### 2.2 Study population

The surgical group comprised parents of children scheduled for elective surgical procedures at the University Children's Clinic in Belgrade. All children scheduled for elective surgical procedures were eligible for inclusion. Eligible parents were consecutively approached and invited to participate during the study period. The inclusion criteria were as follows: all parents of children aged 0–18 years who are scheduled for elective surgical intervention and who have given their written consent to participate in the research, during the research period.

The following criteria excluded participants from surgical group: parents who refuse to give informed consent, parents who are not the biological parents of a child in the hospital, parents who do not understand the Serbian language well, parents of children scheduled for an emergency operation and parents of children who underwent a diagnostic non-surgical procedure under general anesthesia or analgosedation (e.g., magnetic resonance imaging, colonoscopy and gastroscopy).

The non-surgical group consisted of parents of children aged 0–18 years who visited primary health care centers for routine pediatric consultations with selected pediatricians (27) during the same study period (non-surgical pediatric patients), and gave their written consent to participate in the research. Surgical and non-surgical groups were group matched for educational level. In accordance with the Healthcare Law of the Republic of Serbia, children are assigned a selected pediatrician (“chosen doctor”) within the public primary health care (PHC) system. Parents can directly access pediatric services—preventive, diagnostic, and therapeutic—without requiring a referral. Parents who refuse to give informed consent, persons who are not the biological parents of a child and parents who do not understand the Serbian language well were not included in the non-surgical group.

At the outset of the study, all participants received written information stating that participation was voluntary and anonymous, and that all collected data would be confidential. Informed consent was obtained in writing from all respondents prior to participation. Parents in the surgical group completed the questionnaires while waiting for their child's preoperative anesthesia assessment, either a few days before or on the day prior to the scheduled surgical intervention. Parents in the non-surgical group completed the questionnaires while waiting for a routine examination with a primary health care pediatrician. In cases where both parents accompanied the child, only one parent completed the questionnaires, based on mutual agreement.

### 2.3 Data collection

Our study instruments included standardized questionnaires for examining the levels of parental anxiety and health literacy, and a general questionnaire specifically designed for this research. We used the State-Trait Anxiety Inventory (STAI), Form Y (STAI-Y), to assess the presence and severity of anxiety symptoms in our study population. The STAI-Y is a widely used and validated instrument for measuring both situational (state) and dispositional (trait) anxiety. A Serbian-language version of the STAI was used, based on standard translation and cultural adaptation procedures, and previously applied in clinical and research settings. The STAI-Y consists of two subscales, each containing 20 questions. The first segment of the questionnaire (STAI S) measures current anxiety (how someone feels at a given moment), while the second segment of the questionnaire (STAI T) assesses the respondent's usual feelings, independent of the situation in which he is currently. All questions are designed so that the respondent can choose an answer to the offered question on a four-point Likert scale (for usual anxiety from “not at all” to “very much”, while for situational anxiety from “almost never” to “almost always”) (28).

To measure health literacy of our respondents, we used a Serbian version of the Test of Functional Health Literacy in Adults Short Form (STOFHLA). The Serbian version of STOFHLA has been translated and cross-culturally adapted following standard forward–backward translation procedures and evaluated in primary care patients. It demonstrated high internal consistency (Cronbach's *α* = 0.90) and strong correlation with the full Test of Functional Health Literacy in Adults (TOFHLA) instrument (Pearson r = 0.89), confirming its reliability and criterion validity in the Serbian adult population (29). The STOFHLA questionnaire has 36 items selected for the comprehension test, and 4 items selected for the counting test. The respondent has 7 min to complete this questionnaire. The numerical scores and categories for the STOFHLA questionnaire are as follows: inadequate functional health literacy (0–16), marginal functional health literacy (17–22) and adequate functional health literacy (23–36).

In addition, we designed a general questionnaire to collect the basic information about the respondents, including socio-demographic characteristics (gender, age, relationship status, number of children, urban or rural living environment, employment, level of education, profession) and details related to child's surgery. The latter included whether the parents in our surgical group received information about the surgical intervention, potential complications, the child's preoperative and immediate postoperative nutrition, and pain management after surgery. The following information about the child was collected from the medical records: gender, age, diagnosis, and type of hospitalization (one-day surgery or multi-day hospitalization). The nature of the surgery was divided into three categories: minor (procedures lasting less than 1 h and suitable for day-care), intermediate (procedure lasting longer than 1 h) and major (procedures lasting longer than 1 h after which high-dependency care would be appropriate) (30). The preoperative health status of each child scheduled for elective surgery was assessed using the American Society of Anesthesiologists (ASA) Physical Status Classification System, which provides a standardized framework for grading a patient's physical condition before anesthesia and surgery. The ASA score differentiates patients into six categories, ranging from ASA I (a normal healthy patient) to ASA VI (a brain-dead patient whose organs are being removed for donation), while patients classified as ASA IV, V, or VI generally do not undergo elective surgical procedures (31).

### 2.4 Statistical analysis

Data description included absolute and relative numbers in percentages for categorical data and arithmetic mean with standard deviation or median with range for numerical data. Normality was evaluated using mathematical (Shapiro-Willk test) and graphical (histogram and box-plot) methods.

To ensure the reliability of the instrument in this study population, internal consistency and construct validity were assessed. Psychometric characteristics of the STAI-State and STAI-Trait scales were evaluated in both groups of participants (surgical and non-surgical) through next three steps: 1) Response rate is presented as a proportion of acceptance at both scale and each item level; 2) Internal consistency of the STAI-State and STAI-Trait scales was assessed by Cronbach's alpha coefficient, ranging from 0 to 1.0; 3) Exploratory factor analysis was applied in order to evaluate the construct validity of the questionnaires taking into account criteria that must be met [Kaiser-Meyer-Olkin (KMO) statistic and Bartlett's test of sphericity].

The criteria for exploratory factor analysis (EFA) were met in both groups. In the surgical group, the KMO measure was 0.758, and Bartlett's test of sphericity was statistically significant (*p* < 0.001). In the non-surgical group, the corresponding KMO measure was 0.870, while Bartlett's test of sphericity was also statistically significant (*p* < 0.001). Principal component extraction method with the number of factors fixed at two, no rotation, and loading threshold of ≥0.40 was applied.

In order to estimate the possible relationship between STAI-State and STAI-Trait scales we calculated the Spearman`s rank correlation coefficient with the following interpretation regarding the strength of the correlation: the correlation coefficient was interpreted as negligible (<0.1), weak (0.1–0.39), moderate (0.4–0.69), strong (0.7–0.89) and very strong (0.9–1.0) (32).

Regarding the aim of evaluating the relationship between different factors and anxiety measured by STAI-S score in surgery and non-surgery groups linear regression analysis was performed. Firstly, for each categorical variable, dummy variables were created, and each category was evaluated in the univariable analysis. Secondly, all statistically significant variables identified at this stage were considered for inclusion in the multivariable model, while multicollinearity was assessed using the variance inflation factor (VIF). Variables with a VIF > 5 were considered for exclusion from the model (33). In the multivariable model in the group of parents whose children underwent surgery excluded variables according to VIF criteria were the “enough information” and “not enough information”. In same model, in order to avoid coefficient instability, instead of entering all nine individual concern variables that were significant in the univariable analysis into the multivariable model, we created a concern score combining all nine concerns into a single variable. None of the entered variables in multivariable model when analysis the group of parents whose children were not undergoing surgery and attended a pediatrician for a routine health check-up were in collinearity. In both groups, enter regression method was applied.

As the median STAI-S score in our surgical population was 50, corresponding to the previously established and applied cut-off value, anxiety was categorized as lower (≤50) or higher (>50) (6, 63). Thus, in order to describe anxiety levels in our study population, we classified respondents according to the median value of STAI-State score into two classes: with lower and higher anxiety. In this situation, we performed univariable and multivariable binary logistic regression analysis reporting odds ratio (OR), 95% confidence interval of the odds ratio (95% CI OR), and *p* value. All variables that were assessed in this study were tested through univariable analysis. Significant variables from this step were entered into multivariable using the same criterion for assessing multicollinearity as that used for linear regression. The same as for linear modeling, in order to avoid coefficient instability, especially because of the small number of respondents with the outcome of interest, instead of entering all nine individual concern variables that were significant in the univariable analysis into the multivariable model, we created a concern score combining all nine concerns into a single variable. Enter method was applied in surgical, whilst Backward Wald method was in use in non-surgical group because of the small number of respondents with the previously defined outcome.

Confounding variables should not be overlooked, but it is of great importance to distinguish between the factors whose association with the outcome is being investigated and the effects of associated factors. We also took these factors into account and therefore adjusted the final multivariable model in surgery group for parental age over 40 years, another child seriously ill in the past, child age under 5 years, and major type of surgery.

All statistical methods considered significant for the chosen level of 0.05. The analysis was performed in the IBM Corp. Released 2012. IBM SPSS Statistics for Windows, Version 21.0. Armonk, NY: IBM Corp.

Before any analysis, the required minimum sample size for the planned linear multiple regression analysis was calculated. A minimum of 91 respondent was required, assuming a significance level of 0.05, a maximum of five predictors (one per 10 outcomes) in the model, a medium anticipated effect size of 0.15, and a desired statistical power level of 0.8 (34). Also, as STAI-S or STAI-T could be used as independent scales (35–37). The required sample size for their validation following the rule of 5 per item required 100 respondents.

## 3 Results

This study included 98 participants from the surgical group (84% mothers and 16% fathers) and 98 participants from the non-surgical group (74% mothers and 26% fathers).

### 3.1 STAI-S and STAI-T questionnaire psychometric characteristics-response rate, internal consistency and construct validity

A total of 100 parents in both groups were asked to participate in the research, thus the STAI scale response rates were 98% in both groups. Among parents from surgical group the response rate per STAI-S questionnaire items was 100% for 16, 99% for 3, 98% for 1, and 97% for 1 out of 20 questions, while it was 100% for 12% and 99% for 8 out of 20 questions of STAI-T questionnaire. Among parents from the non-surgical group the response rate per STAI-S questionnaire items was 100% for 15% and 99% for 5 out of 20 questions, while it was 100% for 9, 99% for 9, and 98% for 2 out of 20 questions of STAI-T questionnaire. All necessary details regarding item response rate per STAI-T and STAI-S are given in Supplementary Table 1.

Internal consistency of both scales, STAI-State and STAI-Trait, was confirmed in both study groups. The reliability analysis demonstrated excellent internal consistency in the surgical group, with Cronbach's alpha values of 0.90 for both scales. Similarly, in the non-surgical group, Cronbach's alpha was 0.94 for the STAI-State scale and 0.92 for the STAI-Trait scale, also indicating excellent internal consistency. The association between STAI scales was also examined within each group. A moderate positive correlation was found in the surgical group (*ρ*=0.440, *p* < 0.001). In contrast, a strong positive correlation was observed in the non-surgical group (*ρ*=0.838, *p* < 0.001).

Exploratory factor analysis proved the original structure of STAI questionnaire consisting of STAI-State and STAI-Trait scales in both, surgical and non-surgical groups (Table 1). The exploratory factor analysis model revealed two factors in a surgical and non-surgical group, explaining 40% and 48% of cumulative variance, respectively. All 20 items of STAI-State loaded as expected in both groups. One out of twenty (Q14) in a surgical group, and none in a non-surgical group of STAI-Trait did not load as expected. Two out of twenty (Q3 and Q16) in a surgical group, and none in a non-surgical group of STAI-State did not load as expected.

**Table 1:** Exploratory factor analysis of the STAI questionnaire.

Original STAI items	Group			
	Surgical group *n* = 98		Non-surgical group *n* = 98	
	Factor 1	Factor 2	Factor 1	Factor 2
STAI-Trait				
Q1	0.490	−0.309	0.601	−0.218
Q2	0.601	−0.172	0.645	−0.410
Q3	0.481	−0.382	0.663	0.064
Q4	0.563	−0.423	0.719	0.191
Q5	0.520	−0.417	0.663	−0.207
Q6	0.588	−0.572	0.634	0.090
Q7	0.568	−0.370	0.636	0.370
Q8	0.480	−0.396	0.722	−0.233
Q9	0.586	−0.366	0.620	0.318
Q10	0.314	−0.304	0.365	−0.393
Q11	0.344	−0.074	0.567	−0.549
Q12	0.634	−0.504	0.787	0.320
Q13	0.472	−0.017	0.597	0.143
Q14	0.315	0.350	0.671	0.192
Q15	0.594	−0.383	0.712	−0.329
Q16	0.421	−0.325	0.788	−0.131
Q17	0.501	−0.451	0.624	0.398
Q18	0.447	−0.007	0.693	0.248
Q19	0.569	−0.311	0.682	−0.276
Q20	0.559	−0.387	0.750	−0.389
STAI-State				
Q1	0.563	0.208	0.704	−0.288
Q2	0.514	0.060	0.724	0.337
Q3	0.471	0.528	0.772	−0.076
Q4	0.196	0.138	0.236	0.175
Q5	0.445	0.359	0.616	0.315
Q6	0.433	0.317	0.413	0.147
Q7	0.650	0.330	0.699	−0.141
Q8	0.559	0.251	0.681	0.044
Q9	0.572	0.038	0.464	0.421
Q10	0.503	0.492	0.701	−0.282
Q11	0.569	0.153	0.730	0.132
Q12	0.578	0.500	0.588	−0.081
Q13	0.593	0.533	0.738	−0.073
Q14	0.517	0.125	0.486	0.094
Q15	0.534	0.454	0.486	0.161
Q16	0.600	0.558	0.754	−0.189
Q17	0.601	0.229	0.494	0.119
Q18	0.567	0.186	0.553	0.235
Q19	0.473	0.395	0.746	−0.202
Q20	0.658	0.098	0.601	0.326

### 3.2 Characteristics of the surgical and non-surgical groups

We found statistically significant differences between the two groups regarding age distribution, employment status, and place of residence (Table 2). Parents in the surgical group tended to be younger than those in the non-surgical group, with half of the surgical-group parents aged 30–40, while 56% of parents in the non-surgical group were older than 40 (*p* = 0.024). In addition, unemployment was more common among parents in the surgical group than in the non-surgical group (24.5% vs. 10%, *p* = 0.008). As expected, no significant differences were observed in parental educational level between the surgical and non-surgical groups, reflecting the group-matching procedure. Furthermore, urban residence was more common among non-surgical group parents (98% vs. 76.5%, *p* < 0.001), consistent with the different recruitment settings of the non-surgical and surgical groups. A higher proportion of parents in the surgical group reported being satisfied or very satisfied with their lives (with health care services in Serbia) than those in the non-surgical group (55% vs. 36%, *p* = 0.027). Conversely, more families in the non-surgical group reported having another child who had previously experienced serious illness (19% vs. 4%, *p* = 0.006). According to the predefined STOFHLA cut-off values, the mean scores in both groups corresponded to the range of adequate functional health literacy. The average S-TOFHLA score was significantly higher among non-surgical group parents (*p* = 0.044) whilst the distribution of adequate, marginal and inadequate functional health literacy was the same among study groups (*p* = 0.600). In contrast, parents in the surgical group had significantly higher scores on the STAI-State (State-Trait Anxiety Inventory—State) scale (*p* < 0.001).

**Table 2:** Socio-economic and demographic characteristics of surgical and non-surgical groups.

Characteristics	Group		p*
	Surgical group *n* = 98	Non-surgical group *n* = 98	
Parental			
Age, *n* (%)	Valid *n* = 98	Valid *n* = 98	
<30	16 (16.3)	10 (10.2)	**0**.**024**
30–40	46 (46.9)	33 (33.7)	
>40	36 (36.7)	55 (56.1)	
Gender, *n* (%)	Valid *n* = 98	Valid *n* = 98	
Female	82 (83.7)	72 (73.5)	0.082
Male	16 (16.3)	26 (26.5)	
Education level, *n* (%)	Valid *n* = 98	Valid *n* = 98	
Primary	12 (12.2)	5 (5.1)	
Secondary	47 (48.0)	42 (42.9)	0.092
Postsecondary non-tertiary, tertiary, and above	39 (39.8)	51 (52.064.8)	
S-TOFHLA, mean ± sd	29.9 ± 7.4	31.8 ± 5.8	**0**.**044**
Functional health literacy according to S-TOFHLA, *n* (%)	Valid *n* = 98	Valid *n* = 98	
Inadequate (S-TOFHLA 0-16)	7 (7.1)	4 (4.1)	0.600
Marginal (S-TOFHLA 17-22)	5 (5.1)	4 (4.1)	
Adequate (S-TOFHLA 23-36)	86 (87.8)	90 (91.8)	
STAI-S, med (min-max)	51 (25–73)	35 (23–65)	**<0**.**001**
STAI-T, med (min-max)	38 (20–72)	38 (20–68)	0.399
Relationship status, *n* (%)	Valid *n* = 97	Valid *n* = 98	
Single	13 (13.4)	13 (14.0)	0.978
With a partner	84 (86.6)	85 (87.0)	
Employment status, *n* (%)	Valid *n* = 98	Valid *n* = 98	
Employed	74 (75.5)	88 (89.8)	**0**.**008**
Unemployed	24 (24.5)	10 (10.2)	
Place of residence, *n* (%)	Valid *n* = 98	Valid *n* = 97	
Urban	75 (76.5)	95 (97.9)	**<0**.**001**
Rural	23 (23.5)	2 (2.1)	
Perceived household material conditions, *n* (%)	Valid *n* = 98	Valid *n* = 98	
Very bed and bad	5 (5.1)	7 (7.1)	0.831
Mediocre	39 (39.8)	39 (39.8)	
Good and very good	54 (55.1)	52 (53.1)	
Health condition self-assessment, *n* (%)	Valid *n* = 98	Valid *n* = 98	
Very bad, bad, and mediocre	19 (19.4)	18 (18.4)	0.855
Good and very good	79 (80.6)	80 (81.6)	
Parent in a hospital in the past, *n* (%)	Valid *n* = 96	Valid *n* = 98	
Yes (in childhood, in adulthood, both)	58 (60.4)	45 (45.9)	**0**.**043**
No	38 (39.6)	53 (54.13.8)	
Number of children, med (min-max)	2 (1–4)	2 (1–4)	0.715
Another child seriously ill in the past, *n* (%)	Valid *n* = 70	Valid *n* = 73	
Yes	3 (4.3)	14 (19.2)	**0**.**006**
No	67 (95.7)	59 (80.8)	
Another child was in a hospital in the past, *n* (%)	Valid *n* = 70	Valid *n* = 73	
Yes (surgery)	14 (43.8)	18 (50.0)	0.930
Yes (other reasons)	17 (53.1)	17 (47.2)	
Yes (surgery and other reasons)	1 (3.1)	1 (2.8)	
No	38 (54.3)	37 (50.7)	
Perceived satisfaction with health care services in Serbia, *n* (%)	Valid *n* = 97	Valid *n* = 98	
Very unsatisfied and unsatisfied	11 (11.3)	18 (18.4)	**0**.**027**
Neutral	33 (34.0)	45 (45.9)	
Satisfied and very satisfied	53 (54.6)	35 (35.7)	
Children			
Age, *n* (%)	Valid *n* = 98	Valid *n* = 98	
<1 year	15 (15.3)	0 (0.0)	**<0**.**001**
1–5 years	28 (28.6)	18 (18.4)	
5–10 years	32 (32.7)	35 (35.7)	
>10 years	23 (23.5)	45 (45.9)	
Gender, *n* (%)	Valid *n* = 98	Valid *n* = 96	
Female	28 (28.6)	40 (41.7)	0.056
Male	70 (71.4)	56 (58.3)	
Birth order of the child planned for surgery/pediatric examination, *n* (%)	Valid *n* = 98	Valid *n* = 97	
The only child	30 (30.6)	22 (22.7)	0.210
The first out of 2 or more	27 (27.6)	46 (47.4)	**0**.**004**
The second out of 3 or more	21 (21.4)	15 (15.5)	0.283
The middle one out of 3 or more	18 (18.4)	12 (12.4)	0.246
The youngest of 3 or more	2 (2.0)	2 (2.1)	0.992

*for the level of significance of 0.05 according to Chi-square test or Fisher's exact test and Mann–Whitney U test where appropriate.

There was a significant difference in the distribution of children's age categories and birth order between the groups (Table 2). In the surgical group, there were twice as many children aged 0–5 years, half as many children aged over 10 years, and an equal number of children aged 5–10 years compared to the non-surgical group (44% vs. 18%, 24% vs. 46%, and 33% vs. 36%, respectively, *p* < 0.001). Additionally, children of parents from the surgical group were more often the only child in the family, whereas those of parents from non-surgical group were more commonly the first of two or more siblings (*p* = 0.004).

Past and current medical condition of children from both study groups are presented in Table 3. Commonly, children who are scheduled for surgical intervention belonged to ASA class I or class II (79%), were inpatients (64%), with minor or intermediate surgery (45% and 36%, respectively) in urosurgery (32%), orthopedics (21%), general surgery (17%), plastic and reconstructive surgery (16%), cardiac (10%), and neurosurgery (3%). Children scheduled for surgical intervention and non-surgical pediatric patients differed significantly in the prevalence of comorbidities (33% vs. 2%, *p* < 0.001) and congenital disorders (9% vs. 0%, *p* < 0.001). Previous serious medical condition was present in 12% (surgical group) and 18% (non-surgical group) (*p* = 0.008). Previous hospitalization was reported in 53 of 98 children scheduled for surgery and in 63 of 98 non-surgical pediatric patients. Hospitalization due to a surgical procedure was more common among children scheduled for surgery compared to non-surgical pediatric patients (29% vs. 19%, *p* = 0.008).

**Table 3:** Past and current medical conditions of children-surgical and non-surgical group.

Child's Medical History	Group		p*
	Surgical group *n* = 98	Non-surgical group *n* = 98	
Known comorbidities			
Yes	32 (32.7)	2 (2.0)	**<0.001**
No	66 (67.3)	96 (98.0)	
Known congenital disorders			
Yes	9 (9.2)	0 (0.0)	**<0.001**
No	89 (90.8)	98 (100.0)	
History of previous serious ilness			
Yes	12 (12.2)	14 (14.4)	0.653
No	86 (87.8)	83 (85.6)	
History of previous hospitalizations			
Yes (previous surgical procedure)	28 (28.6)	18 (18.8)	**0.008**
Yes, (other reasons)	25 (25.5)	45 (46.9)	
No	45 (45.9)	33 (34.4)	
Type of surgery			
Outpatent	35 (35.7)	/	NA
Inpatient	63 (64.3)	/	
Surgery class			
Minor	44 (44.9)	/	NA
Intermediate	35 (35.7)	/	
Major	19 (19.4)	/	
Surgical specialty of planned procedure			
Urosurgery	31 (31.6)	/	NA
Orthopedics	21 (21.4)	/	
General surgery	17 (17.3)	/	
Plastic and reconstructive surgery	16 (16.3)	/	
Cardiac surgery	10 (10.2)	/	NA
Neurosurgery	3 (3.1)	/	
Physical status of the child			
ASA I	48 (49.0)	/	
ASA II	29 (29.6)	/	
ASA III	21 (21.4)	/	

*for the significance level of 0.05 according to Chi-square test.

The source and amount of information received about the medical procedure the child is scheduled to undergo are presented in Table 4. Most parents whose children were preparing for surgery evaluated the amount of information they received about the intervention as sufficient (83%). Almost all of parents of non-surgical pediatric patients searched information regarding the examination from the primary health care pediatrician (92%), while a half of parents whose children were planned for surgery searched information regarding the intervention from the surgeon scheduled to operate a child (57%). Additionally, nearly 20% of parents in both groups reported obtaining information through informal channels-such as conversations with a physician or nurse they personally knew, discussions with non-medical acquaintances who are parents, or by searching the internet.

**Table 4:** Parental sources and extent of information regarding their child's upcoming surgery.

Variable	Group		p*
	Surgical intervention *n* = 98	Pediatric examination *n* = 98	
**How do you evaluate the amount of information** you received about the need for your child's hospital treatment?**?**			NA
I have too much of information	2 (2.0)	/	
I have enough information	81 (82.7)	/	
I do not have enough information	15 (15.3)	/	
How have you searched for information related to your child's health so far?			
From the selected pediatrician from the Health Center	51 (52.0)	90 (91.8)	**<0**.**001**
From the surgeon who has to operate a child	56 (57.1)	/	NA
Through informal conversations with physicians/nurses I know	19 (19.4)	25 (25.5)	0.304
Through conversations with friends/acquaintances who are not health workers, but have children	16 (16.3)	21 (21.4)	0.361
By searching the internet	22 (22.4)	22 (22.4)	1.000

*for the significance level of 0.05 according to Chi-square test.

The most frequently reported sources of higher anxiety among parents whose children were scheduled for surgery included concerns about the anesthesia itself (73%), the surgical procedure (68%), potential complications related to the surgery (66%), and possible anesthesia-related complications (65%) (Table 5).

**Table 5:** Levels of possible causes of higher anxiety among parents from surgical group regarding their child's upcoming surgery.

Cause of higher anxiety, *n* (%)	Not at all or a little	Moderate	Mostly or absolutely yes
Concerns regarding hospitalization (unfamiliar and new environment)	53 (55.8)	21 (22.1)	21 (22.1)
Concerns regarding organization of arrival and departure from the hospital	53 (55.8)	28 (28.6)	27 (27.6)
Concerns regarding separation from the child	25 (25.7)	18 (18.6)	54 (55.6)
Concerns regarding the surgical procedure	15 (15.3)	16 (16.3)	67 (68.4)
Concerns regarding anesthesia	12 (12.4)	14 (14.4)	71 (73.2)
Concerns regarding surgery-related complications	16 (16.3)	17 (17.3)	65 (66.4)
Concerns regarding anesthesia-related complications	14 (14.3)	20 (20.4)	64 (65.3)
Concerns regarding preoperative diagnostic procedures	59 (60.2)	20 (20.4)	19 (19.4)
Concerns regarding nutrition before and after surgery	58 (59.8)	16 (16.5)	23 (23.7)
Concerns regarding pain that child can experience before and after surgery	20 (20.4)	18 (18.4)	60 (61.2)
Concerns regarding long-term consequences of the surgery	30 (30.6)	20 (20.4)	48 (49.0)
Concerns regarding long-term consequences of anesthesia	31 (31.9)	23 (23.7)	43 (44.4)
Concerns regarding postoperative recovery	24 (24.5)	25 (25.5)	49 (50.0)
Concerns regarding follow-up care after surgery	45 (45.9)	26 (26.5)	37 (37.5)

In order to evaluate the association between parental and children characteristics and anxiety measured by STAI-S score linear regression was performed, in surgery and non-surgery groups. Among parents of children who underwent surgery, those who were satisfied or very satisfied with healthcare had lower anxiety scores, whereas parents of children with an ASA score of 3 and those with a higher overall concern score had higher STAI-S scores (Table 6).

**Table 6:** Factors associated with higher anxiety (STAI score) in parents whose children underwent surgery.

Characteristic	Univariable			Multivariable		
	B	95%CI B	*p*	B	95%CI B	*p*
Healthcare satisfaction						
Satisfied and very satisfied	−4.283	−8.52 to −0.05	0.048	−3.914	−7.61 to −0.22	**0** **.** **038**
ASA 3	5.818	0.75–10.88	0.025	4.959	0.39–9.53	**0**.**034**
Amount of information						
Enough	−7.036	−12.49 to −1.58	0.012			
Not enough amount of information	8.209	2.52–13.90	0.005	5.076	−0.21–10.37	0.060
Concerns regarding separation from the child (mostly)	6.811	2.70–10.92	0.001			
Concerns regarding the surgical procedure (mostly)	8.641	4.40–12.88	<0.001			
Concerns regarding anesthesia (mostly)	9.551	5.09–14.01	<0.001			
Concerns regarding surgery-related complications (mostly)	7.062	2.78–11.34	0.001			
Concerns regarding anesthesia-related complications (mostly)	6.588	2.31–10.87	0.003			
Concerns regarding preoperative diagnostic procedures (mostly)	5.708	0.44–10.98	0.034			
Concerns regarding nutrition before and after surgery (mostly)	5.125	0.17–10.08	0.043			
Concerns regarding pain that child can experience before and after surgery (mostly)	8.560	4.54–12.58	<0.001			
Concerns regarding long-term consequences of the surgery (mostly)	5.460	1.34–9.58	0.010			
Concern score	1.775	1.10–2.45	<0.001	1.471	0.77–1.15	**<0**.**001**

Among parents whose children were not undergoing surgery and attended a pediatrician for a routine health check-up, parents aged 30–40 years and those who reported good or very good household material conditions had lower STAI-S scores (Table 7).

**Table 7:** Factors associated with higher anxiety (STAI score) in non-surgery parents.

Characteristic	Univariable			Multivariable		
	B	95%CI B	*p*	B	95%CI B	*p*
Parental age						
<30	10.632	3.74–17.52	0.003			
30–40	−7.175	−11.56 to −2.79	0.002	−4.948	−9.75 to −0.14	**0**.**044**
Perceived household material condition						
Very bad and bad	8.604	0.30–16.91	0.042	2.009	−9.64–13.66	0.732
Good and very good	−5.775	−9.99 to −1.56	0.008	−6.957	−13.08 to −0.84	**0**.**026**
Self assessed health condition						
Good and very good	−6.646	−11.67 to −1.63	0.010			
Another child seriously ill in the past	−7.858	−14.06 to −1.65	0.014	−3.293	−10.70–4.11	0.378
History of previous serious illness of the child	−6.665	−12.79 to −0.55	0.033	−5.515	−14.06–3.03	0.202

The median and range of STAI-State and STAI-Trait anxiety scores, among parents whose children were scheduled for surgery were 51 (25–73) and 35 (23–65), respectively. Based on a cut-off value of 51 for the STAI-State score, the proportion of parents with higher levels of anxiety in this group was 54%. Among parents of non-surgical pediatric patients, the median and range of STAI-State and STAI-Trait anxiety scores were 38 (20–72) and 38 (20–68), respectively. Using the same cut-off value, the proportion of parents with higher levels of anxiety among parents of non-surgical pediatric patients was 10%.

Finally, we evaluated factors associated with higher level of anxiety among parents from both study groups. Factors associated with higher anxiety among parents whose children underwent surgical procedure are shown in Table 8. Univariate logistic regression analysis established that female parental gender, secondary educational level, insufficient amount of information regarding the need for hospital treatment of the child, obtaining information regarding child's health through conversations with friends/acquaintances who are not health workers, but have children, and concerns about separation from the child, surgical procedure, anesthesia, anesthesia related complications, pain that child can feel before and after the surgery, long-term consequences of the surgery, long-term consequences of anesthesia, postoperative recovery and postoperative control were possibly associated factors with the presence of higher anxiety among parents. Functional health literacy, assessed by STOFHLA, was tested both as a continuous and categorical variable but was not significantly associated with parental higher anxiety and was therefore not retained in the multivariate model. Afterward, multivariable logistic modeling confirmed that primary and secondary educational level (in comparison with higher educational levels) (OR = 6.728, 95%CI OR = 1.31–34.61, *p* = 0.023 and OR = 4.402, 95%CI OR = 1.42–13.63, *p* = 0.01), having insufficient amount of information regarding child`s health (OR = 6.010, 95%CI OR =  1.01/35.90, *p* = 0.049), obtaining information regarding child's health through conversations with friends/acquaintances who are not health workers but have children (OR = 7.286, 95%CI OR = 1.17–45.52, *p* = 0.034), and higher concern score (OR = 1.230, 95%CI OR = 1.03/1.47, *p* = 0.024) were associated factors with the presence of higher anxiety among parents of children who were scheduled for surgical intervention. Additionally, after adjustment for parental age over 40 years, another child seriously ill in the past, child age under 5 years, and major type of surgery, primary and secondary compared to higher educational level and having insufficient amount of information regarding child`s health remained significantly related to higher anxiety among parents in surgery group.

**Table 8:** Factors associated with higher parental anxiety in children scheduled for surgical intervention-a logistic regression analysis.

Factor, *n* (%)	Higher anxiety		Univariate logistic regression		p*	Multivariate logistic regression (Backward: Wald method, Step 7, Log likelihood=90.92)		p*
	Yes *n* = 53	No *n* = 45	OR	95%CI OR		OR	95%CI OR	
Parent gender**								
Male	4 (25.0)	12 (75.0)	Ref.					
Female	49 (59.8)	33 (40.2)	4.455	1.32–15.01	0.016	5.861	1.25–27.53	0.025
Educational level of parents**						5.829	0.98–34.71	0.053
Primary	8 (66.7)	4 (33.3)	3.200	0.82–12.50	0.094	4.459	1.40–14.16	**0**.**011**
Secondary	30 (63.8)	17 (36.2)	2.824	1.17–6.79	**0**.**020**	Ref.		
Postsecondary non-tertiary, tertiary, and above	15 (38.5)	24 (61.5)	Ref.					
The amount of information regarding the hospital treatment of the child**								
I have too much of information	1 (50.0)	1 (50.0)	Ref.			Ref.		
I have enough information	39 (48.1)	42 (51.9)	0.154	0.01–3.58	0.244	2.763	0.14–53.41	0.501
I do not have enough information	13 (86.7)	2 (13.3)	0.143	0.03–0.67	0.014	18.963	0.62–582.25	0.092
Obtaining information regarding child's health through conversations with friends/acquaintances who are not health workers, but have children**								
Yes	14 (87.5)	2 (12.5)	7.718	1.65–36.13	0.009	12.788	1.65–99.23	0.015
No	39 (47.6)	43 (52.4)	Ref.			Ref.		
Concerns regarding separation from the child**								
Not at all or a little	10 (40.0)	15 (60.0)	Ref.					
Moderate	7 (38.9)	11 (61.1)	0.955	0.28–3.30	0.941			
Mostly or absolutely	35 (64.8)	19 (35.20	2.763	1.04–7.33	0.041			
Concerns regarding surgical procedure**								
Not at all or a little	3 (20.0)	12 (80.0)	Ref.					
Moderate	9 (56.3)	7 (43.8)	5.143	1.03–25.60	0.046			
Mostly or absolutely	41 (61.2)	26 (38.8)	6.308	1.62–24.51	0.008			
Concerns regarding anesthesia**								
Not at all or a little	3 (25.0)	9 (75.0)	Ref.					
Moderate	5 (35.7)	9 (64.3)	1.667	0.30–9.16	0.557			
Mostly or absolutely	44 (62.0)	27 (38.0)	4.889	1.22–19.66	0.025			
Concerns regarding anesthesia related complications**								
Not at all or a little	4 (28.6)	10 (71.4)	Ref.					
Moderate	9 (45.0)	11 (55.0)	2.045	0.48–8.77	0.335			
Mostly or absolutely	40 (62.5)	24 (37.5)	4.167	1.18–14.77	0.027			
Concerns regarding pain that child can feel before and after surgery**								
Not at all or a little	4 (20.0)	16 (80.0)	Ref.					
Moderate	10 (55.6)	8 (44.4)	5.000	1.19–21.04	0.028			
Mostly or absolutely	39 (65.0)	21 (35.0)	7.429	2.20–25.09	0.001			
Concerns regarding long-term consequences of the surgery**								
Not at all or a little	11 (36.7)	19 (63.3)	Ref.					
Moderate	10 (50.00	10 (50.00	1.727	0.55–5.48	0.351			
Mostly or absolutely	32 (66.7)	32 (66.7)	3.455	1.33–8.97	0.011			
Concerns regarding long-term consequences of anesthesia**								
Not at all or a little	11 (35.5)	20 (64.5)	Ref.					
Moderate	13 (56.6)	10 (43.50	2.364	0.78–7.14	0.127			
Mostly or absolutely	28 (65.1)	15 (34.9)	3.394	1.29–8.92	0.013			
Concerns regarding postoperative recovery**								
Not at all or a little	7 (29.2)	17 (70.8)	Ref.			Ref.		
Moderate	13 (52.00	12 (48.00	2.631	0.81–8.56	0.108	5.205	1.11–24.35	0.036
Mostly or absolutely	33 (67.3)	16 (32.70	5.009	1.73–14.51	0.003	6.094	1.59–23.39	0.008
Concerns regarding control after surgery**								
Not at all or a little	18 (40.0)	27 (60.0)	Ref.					
Moderate	16 (61.5)	10 (38.5)	2.400	0.89–6.46	0.083			
Mostly or absolutely	19 (70.4)	8 (29.60	3.562	1.29–9.87	0.015			

*for the significance level of 0.05, **there was a statistically significant difference between parents with and without anxiety regarding evaluated factor according to the Chi-square test or Fisher`s exact test (*p* = 0.011, *p* = 0.041, *p* = 0.009, *p* = 0.003, *p* = 0.046, *p* = 0.015, *p* = 0.021, *p* = 0.046, *p* = 0.002, *p* = 0.032, and *p* = 0.039, respectively).

Factors associated with higher anxiety among parents of non-surgical pediatric patients are presented in Table 9. Univariate logistic regression identified that a mediocre level of self-assessed health, presence of a serious medical condition in another child, and a history of serious illness in the child undergoing examination were potentially associated with parental higher anxiety. However, in multivariate analysis, only the presence of a serious medical condition in another child remained associated with lower anxiety levels (OR = 0.063, 95%CI OR = 0.01/0.38, *p* = 0.002).

**Table 9:** Factors associated with higher parental anxiety in non-surgical pediatric patients-a logistic regression analysis.

Factor	Anxiety		Univariate logistic regression		p*	Multivariate logistic regression (Backward: Wald method, Step 3, Log likelihood=35.72)		p*
	Yes *n* = 11	No *n* = 87	OR	95%CI OR		OR	95%CI OR	
Health condition self-assessment**								
Very bad, bad, and mediocre	5 (27.8)	13 (72.2)	4.744	1.26–17.85	**0**.**021**			
Good and very good	6 (7.5)	74 (92.5)	Ref.					
Another child seriously ill in the past**								
Yes	5 (35.7)	9 (64.3)	15.833	0.01–0.38	0.002	15.833	2.66–94.28	**0**.**002**
No	2 (3.4)	57 (96.6)	Ref.					
History of previous serious illness**								
Yes	4 (28.6)	10 (71.4)	4.343	1.08–17.51	0.039			
No	7 (8.4)	76 (91.6)	Ref.					

*for the significance level of 0.05, **there was a statistically significant difference between parents with and without anxiety regarding evaluated factors according to the Chi-square test (*p* = 0.014, *p* < 0.001, and *p* = 0.028, respectively).

## 4 Discussion

In this study, we examined parental anxiety levels and their determinants among parents of children undergoing elective surgical procedures in Serbia, in comparison with a non-surgical group of parents whose children attended routine pediatric examinations. Our results confirm that parental anxiety during the preoperative period is a significant psychological burden, with more than half (54%) of parents whose children were scheduled for surgery experiencing elevated state anxiety levels. This finding is consistent with prior studies reporting high levels of preoperative anxiety among parents, ranging between 44% and 80% (5, 7, 11, 17, 18), although methodological differences and population variations must be considered. In this context, the proportion of parents with higher anxiety levels observed in this study should be interpreted as a descriptive measure. Its value lies primarily in supporting the comparison between groups and in understanding factors associated with higher anxiety levels, rather than as a standalone outcome. Future studies may benefit from including direct measures of perceived need for support, in order to better identify parents who would most benefit from targeted interventions.

The significantly higher STAI-State scores among parents from the surgical group compared to the non-surgical group highlight the emotional toll of anticipating a child's surgery. Parents may internalize anticipated distress of their child, leading to increased emotional arousal even before the procedure takes place. Concerns most frequently reported by parents included fears related to anesthesia, surgical complications, and postoperative pain and recovery—findings that are consistent with previous reports (5, 6). These fears are particularly potent in situations where uncertainty and lack of control are prominent, as is often the case in pediatric surgery. Preoperative anxiety may be amplified by difficulties in accurately appraising medical risk, especially when information is incomplete, overly technical, or inconsistently communicated.

### 4.1 Parental anxiety and its associated factors

In line with the primary aim of the study, this section focuses on factors associated with parental anxiety in the preoperative setting. Our logistic regression analysis revealed several factors independently associated with parental higher anxiety among parents who are waiting for the child's surgery: primary or a secondary (rather than higher) educational level, seeking health-related information through informal sources such as acquaintances who are not healthcare professionals, and experiencing moderate to high concern about the child's postoperative recovery. Among demographic characteristics of parents in the surgical group, multivariate analysis identified only education level as significant, while the child's demographic characteristics were not associated with higher parental anxiety levels.

Female gender emerged as a factor associated with higher anxiety in the preoperative context compared to male gender, which is consistent with other studies (6, 7, 31, 38). Higher anxiety among mothers may be related to greater caregiving responsibilities and emotional involvement during medical interventions (39, 40). Social norms regarding the expression of emotional distress may also contribute to observed gender differences (41).

Primary and secondary education were independently associated with higher anxiety, compared to higher levels of education. Previous studies on the relationship between education level and parental anxiety have reported inconsistent findings, ranging from no association (6, 14, 30, 41), to higher anxiety in less educated (18, 40) or more educated individuals (42), or a U-shaped pattern with higher anxiety at both lower and higher educational levels (10). These heterogeneous findings suggest that the relationship between education and parental anxiety may be influenced by other socioeconomic and informational factors.

Alongside formal education, functional health literacy provides parents with the capacity to understand medical instructions, appraise risks, and communicate confidently with health professionals. Although functional health literacy was predominantly adequate in both study groups, it was not significantly associated with higher parental anxiety. This finding may partly reflect the limited variability in STOFHLA scores in our sample. Moreover, general functional health literacy may not fully capture procedure-specific information needs in the perioperative setting. High levels of functional health literacy among parents have also been reported in other pediatric healthcare settings (43). However, the fact that 15% of parents from the surgical group still reported insufficient information despite preoperative consultations suggests that adequate functional health literacy does not necessarily imply that all perioperative information needs are met. This is consistent with previous findings indicating that even when information is provided, it may not fully meet parents' needs or expectations (17, 44).

Other studies have similarly shown that information deficits or a desire for more information positively correlate with parental anxiety (9, 17, 18, 30). Studies have reported that parents who were thoroughly informed about the upcoming procedure and potential complications experienced less anxiety than those who were not (19, 45). In contrast, Miller and Mangan (46) found that thoroughly informed adult patients reported more tension, depression, and discomfort. These opposing findings may reflect different coping strategies, such as “monitoring,” where individuals seek more information to reduce fear, and “blunting,” characterized by avoidance of excessive information (47). In our study, obtaining health-related information through informal sources was independently associated with higher anxiety (OR = 12.79). However, the very wide confidence interval indicates substantial imprecision of this estimate, and the finding should therefore be interpreted cautiously. Moreover, the direction of this association cannot be established; reverse causality is possible, as parents with higher anxiety may be more inclined to seek information from informal sources. Previous studies also indicate mixed parental preferences regarding informal information: one American study reported that parents did not want to receive treatment-related information from other parents in similar situations (48), whereas another found that nearly one-third of mothers believed that talking to other mothers of anesthetized children would reduce their anxiety (8). These findings highlight the need for further research on the relationship between information sources and parental anxiety and on approaches to preoperative information delivery in pediatric surgery (14, 19, 49).

Interestingly, several specific perioperative concerns were associated with higher anxiety in univariable analyses, including concerns about long-term consequences of surgery and anesthesia and postoperative recovery. Because these individual concerns were closely related, they were combined into an overall concern score for the multivariable analysis, in which a higher overall concern score remained independently associated with higher parental anxiety. These findings suggest that perioperative concerns may warrant particular attention during preoperative communication, although whether addressing these concerns reduces parental anxiety should be evaluated in future intervention studies. In univariable analyses, parental concerns related to separation from their child, anesthesia and surgery, anesthetic complications, pain before and after surgery, long-term consequences of surgery and anesthesia, postoperative recovery, and follow-up visits were associated with higher parental anxiety. Psychological literature has long emphasized the importance of separation as a critical factor in healthy child development (50–52). Stressful separation episodes—such as before surgery—can trigger anxiety in both parents and children (53, 54), as also shown in our study. A critical review by Franck and Spencer identified fear of anesthesia as a major contributor to parental anxiety (55), while more recent studies also highlight fear of the surgical procedure as a key concern (14, 56). Systematic reviews and meta-analyses of adult surgical patients confirmed that preoperative anxiety is up to four times more common among patients who fear complications (57, 58). In the study by Ayenew et al. (7), nearly 80% of parents expressed fears related to long-term consequences of surgery and anesthesia, particularly concerns about possible disability in their child. Fear of perioperative pain is among the most frequently cited causes of parental anxiety (7, 14, 30, 48). Concerns about postoperative treatment are supported by studies from the UK and US, while parents in Hong Kong were found to be more anxious about postoperative care than those in Scotland (30, 48). Concerns about follow-up visits were also observed in our study and correspond with findings by Pomicino et al. (6), where such concerns were associated with parental anxiety.

Higher anxiety levels were less frequent in the non-surgical group. In this group, the presence of a serious medical condition in another child was independently associated with lower anxiety levels. Given the small number of participants with this characteristic and the wide confidence interval, this secondary finding should be interpreted cautiously. In univariable analysis, parents who rated their own health as mediocre, compared with good or very good, were also more likely to have higher anxiety, consistent with previous evidence linking poorer parental physical and mental health with parental anxiety (59, 60). However, this association did not remain significant in the multivariable analysis.

Sociodemographic differences between groups add further nuance. Parents in the surgical group were more likely to be unemployed, live in rural areas, and have lower educational attainment—all factors previously linked to higher psychological distress in caregiving settings. At a system level, these findings suggest that socioeconomic disadvantage may amplify parental vulnerability during a child's medical crisis. Conversely, greater satisfaction with health care services among the surgical group may reflect a more intense engagement with the healthcare system and a greater appreciation of the care received in a high-stakes context.

Children in the surgical group were also more likely to be younger (especially under 5 years of age) and to be an only child. These child-related factors may be associated with higher parental anxiety, as parents of very young children may perceive their child as more fragile or less able to cope with separation and stress. Being the only child may also heighten perceived stakes of the surgical intervention.

### 4.2 Psychometric properties of the STAI instrument

In line with the secondary aims, this section presents the evaluation of the internal consistency and construct validity of the STAI instrument. The robustness of these findings is strengthened by the excellent psychometric properties of the STAI used in this study. Internal consistency, as indicated by Cronbach's alpha, exceeded 0.90 across both STAI-State and STAI-Trait scales and in both study groups, reflecting excellent internal reliability. This is consistent with prior validations of the instrument in clinical and non-clinical settings (28, 61). Moreover, exploratory factor analysis supported the construct validity of the instrument, confirming the expected two-factor structure of state and trait anxiety. While a few items from the STAI-Trait scale in the non-surgical group showed lower factor loadings, the cumulative variance explained remained within acceptable limits, and no item misloaded in a clinically significant way. The observed moderate-to-strong positive correlations between state and trait anxiety scores in both groups support theoretical expectations and further validate the scale's use in this context (32). Overall, these results affirm that the STAI was an appropriate and psychometrically sound measure of parental anxiety in our sample.

### 4.3 Limitations

Certain limitations should be considered when interpreting our results, many of which are inherent to the study design. The observational nature of our study does not allow for causal inference. The self-reporting method raises the possibility of response bias. The timing of questionnaire administration was not fully standardized between the study groups, which may have influenced the reported anxiety levels, as anxiety can fluctuate during the preoperative period. There was an imbalance in the representation of certain variables—most participants were mothers, limiting the generalizability of findings to all parents. However, since mothers are more involved in childcare and often accompany children during the perioperative period, our results are representative of the target population. Confounding remains a potential limitation of this study; however, potential confounding was taken into consideration in the statistical analysis. The use of a sample-median cut-off to dichotomize STAI-State scores is another limitation. Larger studies are needed to confirm these findings and provide more precise estimates of the observed associations. While the University Children's Hospital serves patients from across Serbia, the single-center design limits the generalizability of our findings, highlighting the need for multicenter studies.

## 5 Conclusions

This study indicates that parental anxiety is a prevalent and associated with multiple factors in the context of elective pediatric surgery in Serbia. Anxiety levels were higher among parents of children scheduled for surgery compared with those attending routine pediatric examinations (54% vs. 10%). Primary and secondary educational level (in comparison with higher educational levels), having insufficient amount of information regarding child`s health, obtaining information regarding child's health through conversations with friends/acquaintances who are not health workers but have children, and higher concern score were associated with the presence of higher anxiety among parents whose children were scheduled for surgical intervention. In contrast, among parents attending routine pediatric examinations, the only independently associated factor with higher anxiety was the absence of a serious medical condition in another child in the family; however, this secondary finding should be interpreted cautiously.

Identifying these factors associated with parental anxiety may help inform in development of future strategies to provide more effective support to families during the perioperative period, which warrant further evaluation for their potential to reduce parental anxiety. Our findings highlight the potential value of tailored, multifaceted approaches, including enhanced preoperative education delivered in plain and accessible language, individualized counseling, structured guidance on postoperative care, and focused support for parents identified as having higher odds of anxiety in this study, including mothers and parents with lower educational attainment. Also, parents relying on informal information sources may warrant additional attention during anxiety assessment, and emphasize a need for future prospective studies. Given that concern about postoperative recovery showed the strongest association with higher anxiety levels in our study, preoperative consultations should place greater emphasis on recovery-related issues, clarification of expected outcomes, and reassurance. Additionally, fostering open communication, emotional support, and shared decision-making may represent potential strategies for future intervention studies to determine whether they can alleviate parental anxiety and empower families.

In addition to individual-level interventions, system-level strategies could be considered, such as the development of standardized preoperative information materials, training of healthcare staff in parent-centered communication, and closer integration of psychosocial support services within pediatric surgical care.

Finally, future prospective, multicenter studies using validated anxiety thresholds and appropriately adjusted statistical analyses are warranted to confirm the associations identified in the present study. Additionally, future research should explore longitudinal outcomes, including the potential impact of parental anxiety on children's postoperative recovery, pain perception, and behavioral responses, as well as the effectiveness of tailored interventions in diverse cultural and healthcare settings. Carefully designed, evidence-based strategies addressing parental anxiety have the potential to improve the overall surgical experience for families and contribute to better outcomes for children.

## Data Availability

The raw data supporting the conclusions of this article will be made available by the authors, without undue reservation.
