# Supplementary material for: Factors associated with parental anxiety in elective pediatric surgery
Source: Front Med (Lausanne). 2026 Sep 25;13:1903817. doi: 10.3389/fmed.2026.1903817 (PMC13649659; doi:10.3389/fmed.2026.1903817)
Supplement: Supplementary file 1 [file Supplementaryfile1.pdf]

Supplementary Table 1. STAI-Trait and STAI-State questions

Original STAI items	Surgical group n=98				Non-surgical group n=98				
	Not at all	Somewhat	Quite	Very	Not at all	Somewhat	Quite	Very	
**STAI-Trait**									
Q1	19 (19.4)	26 (26.5)	45 (45.9)	8 (8.2)	6 (6.1)	8 (8.2)	48 (49.0)	36 (36.7)	**<0.001**
Q2	4 (4.1)	22 (22.4)	44 (44.9)	28 (28.6)	5 (5.2)	7 (7.2)	41 (42.3)	44 (45.4)	**0.009**
Q3	5 (5.1)	31 (31.6)	34 (34.7)	28 (28.6)	39 (39.8)	341 (41.8)	16 (16.3)	2 (2.2)	**<0.001**
Q4	11 (11.3)	29 (29.9)	29 (29.9)	28 (28.9)	36 (37.1)	33 (34.0)	19 (19.6)	9 (9.3)	**<0.001**
Q5	46 (47.9)	29 (30.2)	16 (16.7)	5 (5.2)	5 (5.2)	20 (20.6)	56 (57.7)	16 (16.5)	**<0.001**
Q6	15 (15.8)	33 (34.7)	29 (30.5)	18 (18.9)	52 (53.1)	30 (30.6)	13 (13.6)	3 (3.1)	**<0.001**
Q7	13 (13.3)	29 (29.6)	28 (28.6)	28 (28.6)	26 (26.8)	34 (35.1)	30 (30.9)	7 (7.2)	**0.001**
Q8	26 (26.5)	29 (29.6)	35 (35.7)	8 (8.2)	3 (3.1)	14 (14.3)	49 (50.0)	32 (32.7)	**<0.001**
Q9	19 (19.4)	39 (39.8)	21 (21.4)	19 (19.4)	59 (60.2)	25 (25.5)	10 (10.2)	4 (4.1)	**<0.001**
Q10	60 (61.2)	23 (23.5)	12 (12.2)	3 (3.1)	13 (13.3)	22 (22.4)	54 (55.1)	9 (9.2)	**<0.001**
Q11	22 (22.4)	26 (26.5)	35 (35.7)	15 (15.3)	3 (3.1)	13 (13.4)	52 (53.6)	29 (29.9)	**<0.001**
Q12	12 (12.2)	33 (33.7)	29 (29.6)	24 (24.5)	39 (39.8)	37 (37.8)	16 (16.3)	6 (6.1)	**<0.001**
Q13	62 (63.9)	22 (22.7)	12 (12.4)	1 (1.0)	57 (58.2)	28 (28.6)	8 (8.2)	5 (5.1)	0.222
Q14	70 (71.4)	20 (20.4)	6 (6.1)	2 (2.0)	41 (41.8)	35 (35.7)	18 (18.4)	4 (4.1)	**<0.001**
Q15	47 (48.0)	21 (21.4)	25 (25.5)	5 (5.1)	2 (2.0)	28 (28.6)	49 (50.0)	19 (19.4)	**<0.001**
Q16	39 (39.8)	36 (36.7)	16 (16.3)	7 (7.1)	4 (4.1)	25 (25.5)	49 (50.0)	20 (20.4)	**<0.001**

Q17	11 (11.2)	25 (25.5)	31 (31.6)	31 (31.6)	30 (30.6)	41 (41.8)	22 (22.4)	5 (5.1)	**<0.001**
Q18	49 (50.0)	27 (27.6)	17 (17.3)	5 (5.1)	53 (54.1)	28 (28.6)	16 (16.3)	1 (1.0)	0.412
Q19	20 (20.6)	34 (35.1)	29 (29.9)	14 (14.4)	2 (2.0)	16 (16.3)	48 (49.0)	32 (32.7)	**<0.001**
Q20	34 (34.7)	29 (29.6)	27 (27.6)	8 (8.2)	2 (2.0)	15 (15.3)	54 (55.1)	27 (27.6)	**<0.001**
**STAI-State**	**Almost never**	**Sometimes**	**Often**	**Almost always**	**Almost never**	**Sometimes**	**Often**	**Almost always**	
Q1	5 (5.2)	16 (16.5)	42 (43.3)	34 (35.1)	2 (2.1)	17 (17.5)	57 (58.8)	21 (21.6)	0.084
Q2	8 (8.2)	71 (72.4)	16 (16.3)	3 (3.1)	18 (18.6)	65 (67.0)	13 (13.4)	1 (1.0)	0.144
Q3	3 (3.1)	12 (12.2)	37 (37.8)	46 (46.9)	1 (1.0)	16 (16.5)	47 (48.5)	33 (34.0)	0.180
Q4	42 (42.9)	38 (38.8)	7 (7.1)	11 (11.2)	38 (39.6)	39 (40.6)	11 (11.5)	8 (8.3)	0.670
Q5	69 (70.4)	25 (25.5)	2 (2.0)	2 (2.0)	57 (58.8)	36 (37.1)	4 (4.1)	0 (0.0)	0.122
Q6	17 (17.5)	39 (40.2)	33 (34.0)	8 (8.2)	14 (14.3)	32 (32.7)	41 (41.8)	11 (11.2)	0.510
Q7	1 (1.0)	15 (15.5)	46 (47.4)	35 (36.1)	2 (2.0)	24 (24.5)	44 (44.9)	28 (28.6)	0.358
Q8	36 (36.7)	51 (52.0)	10 (10.2)	1 (1.0)	42 (42.9)	41 (41.8)	13 (13.3)	2 (2.0)	0.518
Q9	14 (14.3)	47 (48.0)	25 (25.5)	12 (12.2)	18 (18.4)	48 (49.0)	26 (26.5)	6 (6.1)	0.470
Q10	1 (1.0)	9 (9.2)	37 (37.8)	51 (52.0)	2 (2.1)	16 (16.5)	44 (45.4)	35 (36.1)	0.074
Q11	37 (38.1)	51 (52.6)	8 (8.2)	1 (1.0)	47 (48.5)	44 (45.4)	5 (5.2)	1 (1.0)	0.494
Q12	50 (52.0)	41 (41.8)	5 (5.1)	2 (2.0)	32 (32.7)	54 (55.1)	10 (10.2)	2 (2.0)	0.060
Q13	2 (2.0)	13 (13.3)	35 (35.7)	48 (49.0)	2 (2.0)	16 (16.3)	48 (49.0)	32 (32.7)	0.136
Q14	12 (12.4)	23 (23.7)	40 (41.2)	22 (22.7)	6 (6.1)	35 (35.7)	38 (38.8)	19 (19.4)	0.191
Q15	63 (64.9)	29 (29.9)	5 (5.2)	0 (0.0)	46 (47.9)	45 (46.9)	4 (4.2)	1 (1.0)	0.065

Q16	1 (1.0)	13 (13.4)	37 (38.1)	46 (47.4)	5 (5.2)	10 (10.3)	54 (55.7)	28 (28.9)	**0.014**
Q17	23 (23.5)	54 (55.1)	14 (14.3)	7 (7.1)	24 (24.5)	58 (59.2)	15 (15.3)	1 (1.0)	0.195
Q18	41 (42.3)	42 (43.3)	11 (11.3)	3 (3.1)	41 (42.3)	37 (38.1)	18 (18.6)	1 (1.0)	0.391
Q19	1 (1.0)	14 (14.4)	34 (35.1)	48 (49.5)	1 (1.0)	19 (19.6)	46 (47.3)	31 (32.0)	0.102
Q20	11 (11.2)	59 (60.2)	18 (18.4)	10 (10.2)	20 (20.4)	58 (59.2)	17 (17.3)	3 (3.1)	0.093
